# Supplementary material for: Di-(2-ethylhexyl) Phthalate Limits the Lipid-Lowering Effects of Simvastatin by Promoting Protein Degradation of Low-Density Lipoprotein Receptor: Role of PPARγ-PCSK9 and LXRα-IDOL Signaling Pathways
Source: Antioxidants (Basel). 2023 Feb 14;12(2):477. doi: 10.3390/antiox12020477 (PMC9952605; doi:10.3390/antiox12020477)
Supplement: Supplementary file 1 [file antioxidants-12-00477-s001.zip › antioxidants-2162885-supplementary.pdf]

SUPPLEMENTARY MATERIAL

Di-(2-ethylhexyl) phthalate limits the lipid-lowering effects of simvastatin by promoting the protein degradation of low-density lipoprotein receptor: Role of PPARγ-PCSK9 and LXRα-IDOL signaling pathways

Bei-Chia Guo, Ko-Lin Kuo, Jenq-Wen Huang, Chia-Hui Chen, Der-Cherng Tarng, Tzong-Shyuan Lee

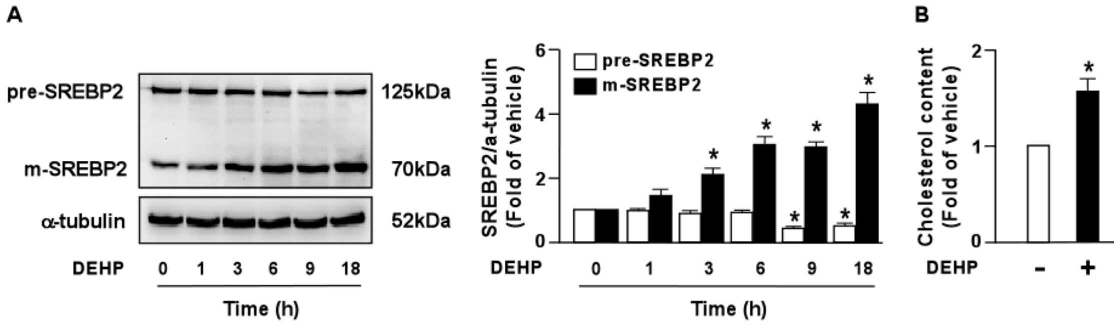

Supplementary Figure 1. DEHP induces SREBP2 protein expression in hepatocytes. **(A)** Huh7 cells were treated with DEHP (1 μg/mL) for the indicated time points (0, 1, 3, 6, 9 and 18 h), and the levels of the precursor of SREBP2 (pre-SREBP2) or mature form of SREBP2 (m-SREBP2) were examined by western blot analysis. (B) The levels of intracellular cholesterol in Huh7 cells. Data are shown as the mean ± SEM from 5 independent experiments. *P < 0.05 vs. vehicle group.
